# Financial incentives to improve uptake of partner services for sexually transmitted infections in Zimbabwe antenatal care: protocol for a cluster randomised trial

**DOI:** 10.12688/wellcomeopenres.19199.2

**Published:** 2023-10-18

**Authors:** Kevin Martin, Chido Dziva Chikwari, Ethel Dauya, Constance RS. Mackworth-Young, Joseph D. Tucker, Victoria Simms, Tsitsi Bandason, Francis Ndowa, Anna Machiha, Sarah Bernays, Michael Marks, Katharina Kranzer, Rashida A. Ferrand

**Affiliations:** 1 Department of Clinical Research, London School of Hygiene & Tropical Medicine, London, UK; 2 Department of Global Health and Infection, Brighton and Sussex Medical School, Brighton, UK; 3 Biomedical Research and Training Institute, Harare, Zimbabwe; 4 Department of Infectious Disease Epidemiology, London School of Hygiene and Tropical Medicine, London, UK; 5 Department of Global Health and Development, London School of Hygiene and Tropical Medicine, London, UK; 6 Skin & Genito-Urinary Medicine Clinic, Harare, Zimbabwe; 7 AIDS and TB unit, Ministry of Health and Child Care, Harare, Zimbabwe; 8 School of Public Health, University of Sydney, Sydney, Australia

**Keywords:** Sexually transmitted infections, partner notification, incentive, point-of-care testing, antenatal care, pregnancy, Zimbabwe

## Abstract

**Introduction:**

Sexually transmitted infections (STIs) such as chlamydia, gonorrhoea, trichomoniasis, and syphilis, are associated with adverse birth outcomes. Treatment should be accompanied by partner services to prevent re-infection and break cycles of transmission. Partner services include the processes of partner notification (PN) as well as arranging for their attendance for testing and/or treatment. However, due to a complex mix of cultural, socio-economic, and health access factors, uptake of partner services is often very low, in many settings globally. Alternative strategies to facilitate partner services are therefore needed.

The aim of this study is to assess the impact of a small financial incentive on uptake of partner services for STIs as part of antenatal care (ANC) services in Zimbabwe.

**Methods and analysis:**

This trial will be embedded within a prospective interventional study in Harare, aiming to evaluate integration of point-of-care diagnostics for STIs into ANC settings. One thousand pregnant women will be screened for chlamydia, gonorrhoea, trichomoniasis, and syphilis. All individuals with STIs will be offered treatment, risk reduction counselling, and client PN. Each clinic day will be randomised 1:1 to be an incentive or non-incentive day. On incentive days, participants diagnosed with a curable STI will be offered a PN slip, that when returned will entitle their partners to $3 (USD) in compensation. On non-incentive days, regular PN slips with no incentive are provided.

The primary outcome measure is the proportion of individuals with at least one partner who returns for partner services based on administrative records. Secondary outcomes will include the number of days between index case diagnosis and the partner attending for partner services, uptake of PN slips by pregnant women, adverse birth outcomes in index cases, partners who receive treatment, and intervention cost.

**Registration:**

Pan African Clinical Trials Registry: PACTR202302702036850 (Approval date 18 ^th^ February 2022).

## Introduction

Curable sexually transmitted infections (STIs) such as chlamydia, gonorrhoea, trichomoniasis, and syphilis, are associated with adverse outcomes in pregnancy ^1^. Unfortunately, globally rates of STIs remain high ^1, 2^. This is particularly true in Southern Africa where control of STIs is hampered by a limited availability of diagnostics, alongside other healthcare access and socio-cultural barriers ^3^.

Partner services are a crucial component of comprehensive case management for an STI in order to prevent re-infection of the index patient. This encompasses both partner notification (PN), whereby a sexual partner of an individual with an STI (the index case) is informed that they may be at risk of an STI, as well as ensuring their attendance for testing and/or treatment ^4^. However, the uptake of PN varies widely, and has not been widely implemented ^5^. When testing for chlamydia and gonorrhoea among young people in community settings in Harare, Zimbabwe, we found that only 8.8% (22/248) of partners returned to sites to receive treatment ^6^. This was using client referral, whereby the individual diagnosed with an STI is counselled and advised to inform their partners to return, often with the aid of a physical PN slip. This is the standard of care in Zimbabwe and many other settings ^7^. However, other strategies such as provider referral and expedited partner treatment have been used in different settings ^4^.

Provider referral refers to a strategy whereby it is the healthcare professional who informs the partner, which may be particularly helpful where the partner in question is a casual partner, whom the index patient may be reluctant to inform. Expedited partner treatment means that medication or a prescription is given directly to the index patient to be given to their partner. This may save time for both the healthcare provider and partner, but may face legal barriers unless specific laws authorising its use are in place ^8^. A systematic review of partner services in sub-Saharan Africa found that the proportion of notified partners who sought evaluation or treatment following client referral was 25% (range 0–77%) compared to 69% for provider referral and 84% for expedited partner treatment. Importantly, the figures for provider referral and expedited partner treatment in this review, were each based on a single study, demonstrating the limited data available to support these strategies ^4^.

Patients’ individual assessment of the risks and benefits of each PN method will vary with context. In antenatal care, one may assume that rates of PN would be better, potentially due to higher proportions of individuals in stable relationships, and the added consideration of ensuring the health of the baby, which may increase indexes’ motivation to inform their partner, and partners’ motivation to attend for treatment. However, several studies in pregnant women reveal a wide range of rates of completion of PN ^4, 9– 12^. This demonstrates that one must consider the broader set of facilitators and barriers to PN that will be unique to each individual. For example, risk of intimate partner violence must be particularly considered in pregnancy, with two-thirds of pregnant women in one study in Harare reporting a history of physical, sexual and/or emotional violence during pregnancy ^4, 13^.

Our experience with young people in Zimbabwe revealed that they found it very difficult to inform partners, and felt ill-equipped to have such conversations with their partners ^6, 14^. In particular, they had genuine concerns regarding their physical safety, with reports of violence noted ^14^. Furthermore, there are also social and emotional risks involved. In addition to potentially precipitating relationship breakdown, accusations of pre- or extra-marital relations may also be disseminated to family, friends or other community members, which may have an impact on an individual’s reputation ^14^. Appropriation of blame and spreading rumours were also raised by patients seeking care in Botswana ^15^. Overall, there was felt to be a “disconnect between the request to notify partners and the reality” of doing so, with the above challenges not being fully acknowledged ^14^. There are also important systems-level barriers to individuals seeking out any STI care, including availability and accessibility of services, with cost being a frequently mentioned barrier to access ^3^. These are all important factors when considering strategies to improve PN uptake. Although higher numbers of partners receiving treatment is optimal from an STI control perspective, individuals must not be exposed to unacceptably high levels of risk in order to achieve this.

This is particularly important for financial incentives, which have the potential to cause both benefit and harm. Incentives have been used in other settings to promote uptake and adherence to various health interventions. This includes improving uptake of HIV testing and result receipt, linkage to HIV treatment and voluntary medical male circumcision, and reducing high-risk sexual behaviour ^16, 17^. Choko *et al.* (2021) investigated the use of partner-delivered HIV self-test kits, with and without financial incentives, in antenatal care in Malawi ^18^. Secondary distribution of test kits substantially increased HIV testing by male partners of pregnant women, by similar magnitudes with and without an incentive. Another study by Choko *et al.* (2018) found that higher proportions of male partners of pregnant women were tested for HIV and linked into care or prevention, when the pregnant woman was provided with two HIV self-test kits for their partners and either a $3 incentive, $10 incentive, or phone reminder. However, no significant increase was noted when HIV self-test kits were provided alone ^19^.

A randomised controlled trial in Harare, Zimbabwe of both fixed and lottery-based incentives given to caregivers showed significantly increased HIV testing uptake among older children and adolescents ^20^. Another trial in rural Zimbabwe also demonstrated that small non-monetary incentives were associated with higher levels of couples’ HIV testing and counselling, and HIV case diagnosis ^21^.

There are fewer examples related to incentives for PN for STIs and a particular paucity of evidence from randomised controlled trials. In a qualitative study in Malawi, healthcare workers at an STI clinic felt that incentivising both partners and couples who attend together would have the greatest effect on improving treatment of partners ^22^. Another study in Malawi used a social contact recruitment programme to recruit social contacts (rather than partners) of individuals with STI syndromes (both with and without HIV), and community controls. Participants (“seeds”) were given coupons to give to their social contacts to come to the clinic for a health promotion visit, including HIV testing and counselling and STI syndromic screening, with seeds receiving $2 for each social contact successfully referred to the clinic. This study demonstrated that this was a feasible, effective and efficient method to identify individuals with undiagnosed HIV ^23^. 

Overall, incentives to improve uptake of PN for STIs have received insufficient attention to establish a clear evidence base regarding their use.

### Rationale

Partner notification has very low take-up in many settings globally. Alternative strategies to facilitate both index patients informing their partners, and particularly partners attending for treatment, must be considered to reduce reinfection, particularly for pregnant women. Incentives, financial or non-financial, to facilitate PN should be considered as a possible strategy. This approach has been used to promote uptake of other health interventions and could be programmed within services especially as it focuses on achieving a discrete outcome rather than requiring repeated engagement over time.

The rationale behind this intervention is that providing a financial incentive may specifically ameliorate some of the socioeconomic barriers that partners face in attending clinic for treatment, in particular transport costs. Importantly, it is not expected that this intervention will significantly influence the major barriers present for index cases to notify their partners in the first instance.

The aim of this study is to assess the impact of a small financial incentive on uptake of partner services for sexually transmitted infections within antenatal care services in Zimbabwe. It will also inform future studies related to partner services, in terms of feasibility and operationalisation of incentives.

## Protocol

### Study design and setting

A cluster randomised trial will be embedded within the “IPSAZ study” (Investigating point-of-care diagnostics for sexually transmitted infections and antimicrobial resistance in antenatal care in Zimbabwe), a prospective interventional study being conducted in urban primary healthcare clinics (PHCs) in Harare province, Zimbabwe, aiming to evaluate a strategy for integration of point-of-care diagnostics for STIs into ANC settings ^24^. Such PHCs provide routine nurse-led antenatal care.

### Study population and recruitment

Pregnant women will be consecutively enrolled into the IPSAZ study when attending a study clinic for routine ANC. The only exclusion criteria are prior enrolment into the IPSAZ study, or being unable or unwilling to provide written informed consent.

### Main study procedures

The procedures for the main IPSAZ study are described in detail in the main study protocol ^24^. In summary, in addition to HIV and syphilis testing provided as part of routine care, the IPSAZ study will provide on-site opt-out testing for chlamydia and gonorrhoea using Xpert® CT/NG assay (Cepheid), for trichomoniasis using OSOM® Trichomonas Rapid Test (Sekisui Diagnostics), and for Hepatitis B using the HBsAg 2 (Abbott Diagnostics Medical Co. Ltd). Comprehensive case management, including treatment and partner notification, will be provided as per national guidelines ^7^, ideally on the same day as sample collection. For participants unable to be treated on the same day, they will be contacted by telephone up to five times over 28 days, to advise them to return for treatment. For participants with symptoms, participants will be given the option to receive immediate syndromic treatment, or to receive tailored treatment following processing of their results.

All pregnant women will be contacted by telephone after birth, to collect data on birth outcomes. Alongside this, they will be asked if they notified their partners, if their partners were treated, and if there were any negative consequences of this, including verbal, physical, sexual, or other abuse, relationship breakdown, negative reactions from friends or other family members, or any other ramifications.

### Proposed intervention

As per standard of care, partners will be notified by client referral. Post-test counselling to participants diagnosed with an STI or treated for a syndrome will include the importance of partner treatment. They will be provided with PN slips for their partners to return for presumptive treatment. Index cases can receive as many PN slips as they require. Partners who attend the study clinic and present their PN slip will be provided with presumptive treatment free-of-charge. Partners will be considered lost-to-follow up if they have not returned within 28 days after treatment of the index case.

The intervention will be provision of $3 (USD) provided to a partner on returning to the clinic for treatment. The amount was based on in-depth interviews with pregnant women, male partners, and midwives, recruited when attending or working at one of the intervention clinics, as well as discussions with members of the intervention team. Questions on appropriate incentive value were embedded within broader discussions about PN, including different methods of PN, associated challenges, and feasibility of PN. Specifically for incentives, stakeholders were asked what they thought about the idea, any potential negative consequences, and what a suitable value would be for an incentive. Male partners often noted that an incentive should cover cost of travel, but also leave the partner with some additional money to spend. In contrast, pregnant woman and midwives tended to say any financial incentive, even very small, would prompt partners to attend. They also noted that unintended consequences could include using the incentive for alcohol or drugs, or for individuals who were not the index’s real partner to attend. A compromise value of $3 was therefore chosen, with a key factor being that $3 covers a return journey by public transport from the majority of locations from where pregnant women (and therefore potentially their partners) are likely to travel.

### Randomisation and blinding

Randomisation of each clinic day will be performed in a 1:1 ratio between days where issued slips are associated with an incentive and days where no incentive is issued. Randomisation by clinic day was chosen as a method to allocate incentives, in preference to individual randomisation, to prevent contamination. Additionally, if some individuals presenting on the same day were offered an incentive and others were not, this may lead to a greater perception of unfairness amongst participants.

Randomisation will be performed by a statistician not involved in the IPSAZ study, using the formula “=INT(RAND()*2)” in Microsoft Excel for each clinic day. Randomisation outcomes will be recorded on a document produced for each month, and kept at each clinic site. The intervention team will refer to this document to determine whether it is an ‘incentive’ or ‘non-incentive’ day. For each individual pregnant woman, it will also be documented whether an issued PN slip was associated with an incentive or not. Similarly, when partners return it will be recorded if an incentive was provided. To ensure compliance with the randomisation log, this data will regularly be cross-checked to ensure that clients are being provided the correct slip based on day of attendance.

It is not possible to blind participants or researchers to the intervention. However, participants will only be informed of the availability of an incentive once they test positive on an incentive day and have already informed the clinical team how many PN slips are required. This is to prevent participants from providing an artificially elevated number of partners in order for incentives to be provided to individuals who are not actual partners of the index case. Of note, women could still return with a male who is not a sexual partner, and this would be difficult to detect. However, given the factors discussed regarding ensuring the health of the baby, and that this would still require disclosure of treatment to another male, we feel that the risk of this is low.

### Outcomes

The primary outcome measure is the proportion of indexes with at least one partner who returns to the study site for partner services within 28 days of index diagnosis. Each PN slip will also have a unique ID that links partners to the index client, thus allowing for recording of this data. The main secondary outcome will be the number of days between index case diagnosis and the partner attending for partner services. Additional secondary outcomes will include uptake of PN slips by pregnant women, adverse birth outcomes in index cases, number of partners who receive treatment, and intervention cost. Number of partners who receive treatment will be recorded from our study antibiotic administration records, and compared with that reported by participants during telephone follow-up.

Furthermore, the proportion of indexes with at least one partner who returns to the study site for partner services any number of days from index diagnosis, will also be measured as a secondary outcome.

### Sample size calculations

As a trial embedded within a larger study, sample size calculations were not performed to power this trial. The main IPSAZ study has a target sample size of 1000 pregnant women, based on an estimated composite STI prevalence of 30%, a desired precision of 3%, an alpha of 0.05, and an additional 10% of participants to account for invalid test results ^6, 25– 33^.

From the initial pilot data from the IPSAZ study, we estimated a 30% prevalence of curable STIs and a recruitment rate of 5 participants per clinic day. For analysis, each clinic day will be considered as a unit of randomisation. It is therefore predicted that 300 index participants will receive partner notification slips, over 200 recruiting clinic days. This equates to 100 ‘clusters’ per arm, with an average of 1.5 participants per cluster.

The intra-cluster correlation coefficient (ICC) is assumed to be zero as the outcome is not expected to be more or less likely in participants having attended on the same day, compared to on different days. However, given the very small size of the clusters, even if the ICC was higher, it is unlikely to have a meaningful effect on the design effect size.

Initial data suggest that an estimated 30% of indexes have at least one partner returning for treatment. Assuming this, and an alpha of 0.05, a power of 0.8, an ICC of zero, 100 clusters per arm, and 1.5 participants per cluster, results in a minimum detectable odds ratio of 1.96 for the intervention ( Table 1). A higher ICC of 0.10 results in a minimum detectable odds ratio of 1.99.

**Table 1.** Minimum detectable odds ratios for effect size of intervention, for differing proportions meeting the primary outcome in the control arm, assuming an alpha of 0.05, 100 clusters per arm, and 1.5 participants per cluster.

		ICC = 0.0		ICC = 0.1	
Sample size	Proportion meeting primary outcome in control arm	Smallest odds ratio detected at 80% power	Smallest odds ratio detected at 90% power	Smallest odds ratio detected at 80% power	Smallest odds ratio detected at 90% power
300	20%	2.09	2.33	2.13	2.37
	25%	2.01	2.22	2.04	2.27
	30%	1.96	2.17	1.99	2.21
	35%	1.93	2.13	1.96	2.17
	40%	1.92	2.12	1.95	2.16

### Statistical analysis

Simple point estimates for each arm will be presented by calculating the number of participants given a PN slip for whom at least one partner returned to the study clinic for treatment divided by the total number of participants given a PN slip. This was chosen over a mean of cluster responses, due to the higher importance of giving equal weight to each individual, over that of each cluster. Analysis will be by intention-to-treat. Analysis per protocol will also be conducted as a sensitivity analysis, based on the type of PN slip that was recorded as being given.

Trial arms will be compared using individual-level logistic regression, using robust standard errors to account for any clustering. Robust standard errors will be used, over generalised estimating equations or random effects models, as the expected correlation within clusters is minimal. Results will be presented as per the CONSORT extension for cluster trials ^34^.

### Process evaluation

An accompanying mixed methods process evaluation will be conducted to further understand the PN process and the influence of incentives on uptake, within the broader IPSAZ study process evaluation, described in the main study protocol ^24^. This process evaluation follows the MRC process evaluation framework, with research domains including fidelity, coverage, responses to and interactions with the intervention, interactions and consequences, and context. Key aspects relevant for PN and this trial are feasibility, acceptability to both pregnant women and partners, how incentives influence interactions between the index case and their partners, and unanticipated pathways or consequences. Of note, assessments of acceptability will explore comparisons between standard PN, incentivised PN, and also no PN, which may be the most acceptable option for some participants. Methods include; in-depth interviews and focus group discussions with pregnant women, healthcare workers, members of the intervention team, and male partners; unstructured observations; and routine monitoring data. Unstructured observations will be of clinical encounters and will be conducted by a research assistant not involved with delivery of the intervention. As observation may impact consultations, permission will be requested from participants, confidentiality will be re-assured, and the research assistant will aim to be as unobtrusive as possible. Interviews with pregnant women and partners will be conducted after PN has been attempted or completed, in order to assess for unintentional consequences or adverse outcomes. As previously mentioned, negative consequences of PN will be documented during a post-natal telephone call.

### Data management procedures

Data management procedures for this incentives trial are the same as for the main IPSAZ study, and are described elsewhere ^24^.

### Ethics and dissemination

Ethical approval for the IPSAZ study protocol, including the incentives trial, has been provided by the Medical Research Council of Zimbabwe (MRCZ/A/2899), the London School of Hygiene & Tropical Medicine Research Ethics Committee (26787), and the Biomedical Research and Training Institute Institutional Review Board (AP176/2022).

Written informed consent to participate in the main IPSAZ study will be obtained in either English or Shona, depending on participant preference. This will include from pregnant minors, who are considered emancipated in Zimbabwe.

Importantly, although participants will be counselled on the benefits of PN, it is recognised that participants may have valid concerns about disclosing this information to partners, in terms of risk of relationship breakdown or intimate partner violence. As a result, we will support participants in coming to their own decision on whether to inform their partner or not.

Adverse events will be documented and discussed at regular debrief sessions. This will include any instances of harm to either indexes or their partners, as a result of the partner notification process. This includes some of the potential negative consequences that we will routinely collect from participants during follow-up, including verbal, physical, sexual, or other abuse. For participants reporting abuse, the needs of the participant will be discussed as part of a multi-disciplinary team at the clinic. Referral processes will be integrated into existing clinic processes as much as possible. Possible onward referrals include to governmental sexual and gender-based violence clinics, charitable organisations focussed on intimate partner violence, counselling services, and/or the police, if required. Additionally, if the participant is under the age of 18 years, referral to social services will be considered. 

Adverse events may also be reported to the Medical Research Council of Zimbabwe if warranted, for example due to severity.

Results will be submitted to open-access peer-reviewed journals, presented at academic meetings and shared with participating communities and with national and international policy-making bodies.

## Discussion

There has been a significant push towards development and integration of new diagnostics for STIs into health systems in the Global South. Importantly however, without concurrent improvements in the key tenets of STI management, namely risk reduction counselling, condom use, and effective partner notification and treatment, the potential benefits of aetiological diagnosis may be limited. One strategy that may help to support partner services is the use of incentives, which may be financial or non-financial. In both instances, the aim is to nudge the partner so that they are more likely to attend a clinic for treatment. However, particularly for financial incentives, they may also improve the likelihood of an index patient informing a partner, if it makes them feel more able to deliver ‘bad news’ if accompanied by something beneficial.

We have chosen $3 (USD) based on input from key stakeholders, from an initial potential range of $1 to $10. An important factor in choosing $3 was that we hoped this would facilitate partners to attend, but not induce them. From an ethical perspective, there is a risk of coercion with larger incentives in socioeconomically deprived communities. Given that PN may carry a risk of violence or relationship breakdown, it is important that the incentive does not force women to inform their partners due to the size of the incentive, against their better judgement ^6^. Importantly, as the direct beneficiaries of the incentive are the partners, we anticipate this is less likely compared to if the incentive was directly benefitting the index. Additionally, it will be emphasised to the intervention team that pregnant women should be supported in coming to the right decision for them, which may be not notifying their partner if there is a risk of negative repercussions.

A further consideration is that higher incentives, although likely to be more effective in research settings, are less likely to be implemented in practice in resource-limited health settings. A one-off payment of $3 could be a programmable intervention, and also be more equated to a ‘nudge’ compared to $5 or $10 which are more akin to direct payments. If found to be successful, other mechanisms to enhance sustainability will need to be considered. For example, they could be used as an entry point for HIV testing for this high-risk group, or other interventions.

Another important decision was the PN strategy to be used. There is currently no consensus on the most appropriate PN strategy, and ideally this should be tailored to the particular setting ^5, 35^. Client referral will be used in this study, largely due to what is likely to be feasibly implemented in routine practice in the future. In Zimbabwe, chronic staff shortages make more resource-intensive PN strategies much less likely to be implemented.

Importantly, although a shift to aetiological testing will bring a host of advantages to STI management in Southern Africa, it will also bring some additional challenges. Given that syndromic management has been in place for decades, a cultural shift will be required for both clients and healthcare workers, towards an understanding of asymptomatic infections, and the need for screening and treatment. This may have ramifications for PN, with the potential for reluctance if neither index patient not partner have symptoms. This was raised in a mixed methods study in Gaborone, Botswana, where a lack of symptoms was noted as a barrier for index cases to inform their partners, as they did not think their partner had an infection ^15^. The difficulty of explaining that someone might have an infection without symptoms was raised. Furthermore, general trust in health systems and health providers will also inform these decisions. Incentives and other methods to promote PN will therefore be important in facilitating this shift.

A key strength of this study is that the intervention has been tailored and informed based on input from key stakeholders. Additionally, although sample size calculations were not performed with this trial in mind, it will likely be moderately powered to detect clinically significant differences in PN uptake.

Important limitations relate to generalisability. Firstly, this study will be conducted in urban PHCs in Harare. Results are likely less generalisable to rural settings, where distance to nearest clinic, availability of transport, expected quality of care, and socioeconomic differences, may alter the perception of such an incentive. Secondly, this is an ANC study, which is unique for several reasons; 1) All indexes will be pregnant women; 2) most if not all partners will be male; 3) the health and wellbeing of the baby will likely be an important factor in decision-making; and 4) there may be a higher proportion of stable relationships compared to other settings. These unique circumstances mean that any attempts at extrapolating these findings to other settings or sub-populations must be done so with a high level of caution.

A further limitation is that our primary outcome is based upon at least one partner returning for treatment. This does not take account of index cases with more than one sexual partner. However, demographic health and survey data from Zimbabwe reported that less than 1% of surveyed women and 2.5% of surveyed men reported concurrent sexual partners in the preceding 12 months ^36^. Furthermore, as data on both number of partners and number of partners treated will be collected, we will be able to make an assessment of the degree to which partner notification was successful in index cases with multiple partners ^37^.

Finally, all clients and their partners receive treatment free of charge in this study, which is not representative of clinic settings in Zimbabwe, where fees and co-payments are standard. This may therefore influence how we interpret differences between groups, as even in the control arm, an important barrier to access has already been removed. Additionally, as treatment is available from most PHCs, there is a risk that partners may attend the PHC most convenient to them, and thus not be included as a study outcome. However, providing treatment free of charge in the control arm will reduce this risk.

As far as we are aware, this is the first randomised controlled trial to assess the effect of a small financial incentive on PN for STIs. It will provide important data on the potential for the use of financial incentives to improve uptake of PN in urban, Southern African settings. Although it will not replace the need for cohesive and funded PN strategies tailored to the population served, incentives may enhance baseline uptake, allowing for reduced rates of re-infection and their associated complications.

## Data Availability

No data is associated with this article. Following publication of study results, the subset of data required for the purposes of verifying research findings will be made available for sharing and will be placed in Data Compass (the London School of Hygiene & Tropical Medicine institutional research data repository—accessible at https://datacompass.lshtm.ac.uk/). This repository will enable direct download of records with codebooks to enable replication of the data analyses. A more complete sharing of data with any research group requesting access to individual data records will be done 12 months after publication. At this point, all data and study tools will be made available through Data Compass. Data for sharing will be de-identified prior to release. Details of how to access data will be published with each study publication.
