# Supplementary material for: Financial incentives to improve uptake of partner services for sexually transmitted infections in Zimbabwe antenatal care: protocol for a cluster randomised trial
Source: Wellcome Open Res. 2023 Oct 18;8:263. Originally published 2023 Jun 21. [Version 2] doi: 10.12688/wellcomeopenres.19199.2 (PMC10521034; doi:10.12688/wellcomeopenres.19199.2)
Supplement: Peer review history

# Reviewer report: Reviewer response for version 2

**DOI:** 10.21956/wellcomeopenres.22400.r68892

**Published:** 2023-11-21

**Authors:** Jana Jarolimova

**Affiliations:** 1 Massachusetts General Hospital, Boston, Massachusetts, USA

Thank you to the authors for addressing the reviewers' comments. I have reviewed the revised version and have no further comments.

Is the study design appropriate for the research question?

Yes

Is the rationale for, and objectives of, the study clearly described?

Yes

Are sufficient details of the methods provided to allow replication by others?

Yes

Are the datasets clearly presented in a useable and accessible format?

Not applicable

Reviewer Expertise:

Implementation science; epidemiology; sexually transmitted infections

I confirm that I have read this submission and believe that I have an appropriate level of expertise to confirm that it is of an acceptable scientific standard.

# Reviewer report: Reviewer response for version 2

**DOI:** 10.21956/wellcomeopenres.22400.r68895

**Published:** 2023-11-01

**Authors:** Augustine Choko

**Affiliations:** 1 Malawi Liverpool Wellcome Trust Clinical Research Programme, Blantyre, Malawi; 2 International Public Health, Liverpool School of Tropical Medicine, Liverpool, UK

I have reviewed this revised version and I have no further comments.

Is the study design appropriate for the research question?

Partly

Is the rationale for, and objectives of, the study clearly described?

Yes

Are sufficient details of the methods provided to allow replication by others?

Yes

Are the datasets clearly presented in a useable and accessible format?

Not applicable

Reviewer Expertise:

NA

I confirm that I have read this submission and believe that I have an appropriate level of expertise to confirm that it is of an acceptable scientific standard.

# Reviewer report: Reviewer response for version 1

**DOI:** 10.21956/wellcomeopenres.21280.r63561

**Published:** 2023-09-15

**Authors:** Mathildah Mokgatle

**Affiliations:** 1 Sefako Makgatho Health Sciences University, Pretoria Ga-Rankuwa, South Africa

Review of the protocol on **Financial incentives to improve uptake of partner services for sexually transmitted infections in Zimbabwe antenatal care: protocol for a cluster randomized trial**

 Thank you for the opportunity to review the protocol which aims to assess the effect of a small financial incentive on improving PN for sexually transmitted infections within antenatal care services in Zimbabwe.

 The proposal is nested in a broad study which is investigating point-of-care diagnostics for sexually transmitted infections and antimicrobial resistance in antenatal care in Zimbabwe. The proposed design is a clustered randomized trial and the clustered are structured according to randomizing the alternative days of incentive given and no incentive given. 

 The study will use the existing healthcare systems guideline on the management of STI where a partner notification slip is used to the patient who is the index case, then the slip gets delivered to the sexual partner for use to access STI treatment. Index cases can receive as many PN slips as they require. Partners who attend the study clinic and present their PN slip will be provided with presumptive treatment free-of-charge.

 The intervention will be provision of $3 (USD) provided to a partner on returning to the clinic for treatment. 

 The proposal is methodologically sound and covers all gaps required to enable replicability. 

 I have only one concern that can easily be addressed:

 The researchers say that **Partners will be considered lost-to-follow up if they have not returned within 28 days after treatment of the index.**

 The concern is that the reality is that not all sexual partners are able to use the health facility that is used by the index case. I read about possible risks for the study of which one of them is patient following different pathways? I wonder if this will accommodate the loss-to-follow up but I would prefer the researchers to put it clearly in order to strengthen the section of partner lost-to-follow up.

 Overall the protocol is elaborate and comprehensive and will be useful for replication regarding feasibility and operationalization of incentives.

Is the study design appropriate for the research question?

Yes

Is the rationale for, and objectives of, the study clearly described?

Yes

Are sufficient details of the methods provided to allow replication by others?

Yes

Are the datasets clearly presented in a useable and accessible format?

Not applicable

Reviewer Expertise:

Social epidemiology and interventions for sexual and reproductive health across populations.

I confirm that I have read this submission and believe that I have an appropriate level of expertise to confirm that it is of an acceptable scientific standard.

# Reviewer report: Reviewer response for version 1

**DOI:** 10.21956/wellcomeopenres.21280.r61808

**Published:** 2023-08-18

**Authors:** Sonali Wayal

**Affiliations:** 1 University College London, London, UK

The manuscript is well written and describes the proposed intervention, its rationale and evaluation strategy clearly. The manuscript can benefit from clarifying the following issues:

1. Brief explanation about how the randomisation by clinic day will be implemented, specifically how will it be ensured that staff will offer the correct PN strategy for the allocated clinic day as this can have implications for contamination 
2. Double check the OR outlined in the table 1 and the estimated minimum detectable OR in the text. 
3. Clarify as part of process evaluation what structured and unstructured observations will be conducted, by whom and its ethical implications if any.
4. Telephone calls will be conducted post birth when participants will be asked about negative impact if any of PN. Besides reporting to MRC, please address clearly what measures will be in place to address negative effects of PN among participants of both the study arms and especially intervention arm. 
5. The protocol has estimated for the minimum number of sexual partners who will return for treatment. it will also be useful to briefly discuss the nature of sexual partnerships in Zimbabwe (for ex: monogamous, polygamous, married/unmarried etc) and its potential implications on outcomes especially as there is growing evidence that nature of sexual partnerships has implications on PN outcomes.
6. It will be useful to briefly outline the theory of change informing the proposed intervention and anticipated levers that will facilitate behaviour change. currently this information is scattered throughout the manuscript in separate sections and can benefit from clarifying the anticipated behaviour change pathway. 

Best wishes

Is the study design appropriate for the research question?

Yes

Is the rationale for, and objectives of, the study clearly described?

Yes

Are sufficient details of the methods provided to allow replication by others?

Yes

Are the datasets clearly presented in a useable and accessible format?

Not applicable

Reviewer Expertise:

Sexual partnerships, partner notifications, bacterial STI and HIV, development and evaluation of behavioural interventions.

I confirm that I have read this submission and believe that I have an appropriate level of expertise to confirm that it is of an acceptable scientific standard, however I have significant reservations, as outlined above.

# Reviewer report: Reviewer response for version 1

**DOI:** 10.21956/wellcomeopenres.21280.r61815

**Published:** 2023-08-18

**Authors:** Augustine Choko

**Affiliations:** 1 Malawi Liverpool Wellcome Trust Clinical Research Programme, Blantyre, Malawi; 2 International Public Health, Liverpool School of Tropical Medicine, Liverpool, UK

It is a real pleasure to be able to review this protocol for a CRT in Zimbabwe using a $3 financial incentive to increase partner clinic attendance following an STI diagnosis of an index. The authors have articulated very clearly multiple barriers to clinic attendance in the target population but also more generally. What might be more useful is a justification and a reflection as to why the offer of a $3 financial incentive conditional on clinic attendance might address all the barriers cited. In other words, some thought on the mechanism of how the intervention might produce the intended effect would be more important and would provide rigour to the argument of incentives as an intervention. 

 The choice of the clinic day as a cluster is very interesting and one that has been used successfully to reduce time in another similar study in Malawi. It might be more helpful, though, to pay more attention on the small nature of the cluster, here the authors state number of eligible participants per clinic day of as few as 1 participant. This raises serious problems a) with respect to the use of a cluster randomized trial design whose basis is that there is variation within and between clusters. Such an argument of variation may not apply here if the cluster is largely made of size 1 b) even if a CRT were to apply, there is extremely high likelihood that many such small clusters will produce a zero participant achieving the outcome. This needs a lot of thought at the outset as it implies that standard CRT methods of analysis may not hold. Other more complex i.e. zero inflated models or methods may thus have to be used or considered.

 I would like to ask the investigators to reconsider the implementation of the study on the basis that it seems already highly underpowered. They state that no study power can be computed because the overall sample size is fixed by the parent study. This is a little puzzling because as CRT with an intervention component it is imperative to compute and demonstrate that the trial will have sufficient power to detect a difference in the primary outcome at least. In the absence of such a calculation I feel that it may be worthwhile conducting this as an exploratory study as opposed to a full scale CRT.

 The assumption of a zero ICC may need additional thought. While there may be little no variation within clinic days at any one given clinic, this may not be the case between health facilities between clinic days. Choko et al PLOS Med 2019 still assumed k of 0.10 under very similar circumstances in Malawi and their analyses showed that ICC was not equal to zero. This implies that the stated sample size of 100 cluster per arm is smaller than the correct number of clusters per arm i.e. the true required number of clusters per arm will be larger.

Is the study design appropriate for the research question?

Partly

Is the rationale for, and objectives of, the study clearly described?

Yes

Are sufficient details of the methods provided to allow replication by others?

Yes

Are the datasets clearly presented in a useable and accessible format?

Not applicable

Reviewer Expertise:

HIV testing, linkage to prevention and treatment, cluster randomized trials

I confirm that I have read this submission and believe that I have an appropriate level of expertise to state that I do not consider it to be of an acceptable scientific standard, for reasons outlined above.

# Reviewer report: Reviewer response for version 1

**DOI:** 10.21956/wellcomeopenres.21280.r61820

**Published:** 2023-08-09

**Authors:** Jana Jarolimova

**Affiliations:** 1 Massachusetts General Hospital, Boston, Massachusetts, USA

This protocol is for a randomized controlled trial evaluating the impact of a financial incentive on partner notification and partner treatment for curable sexually transmitted infections diagnosed among pregnant women attending antenatal care in urban Zimbabwe. It is nested within a prospective interventional study of etiologic screening and treatment of STIs in pregnancy. Pregnant women diagnosed with a curable STI will be randomized, by day, to either standard partner notification procedures by client referral (with free treatment for the partner) or to a small financial incentive for each partner referred and treated. The primary outcome is the proportion of index patients with at least one partner who presents to the study clinic for treatment within 28 days.

**General comments:** Overall, this is a clearly written protocol. However, several details of the methods can use clarification and the discussion of the complexities of partner notification could be expanded upon as below:

- The discussion about the potential harms of partner notification for the index patient (particularly for female index patients) in both the introduction and discussion is underdeveloped. There is a growing body of literature on facilitators and barriers to partner notification, including from Southern Africa. It would be helpful to more clearly describe what has been identified as barriers in both the authors’ recent work and elsewhere in the literature and then to describe which of those barriers the financial incentive may help to overcome.
- A more nuanced discussion of how the barriers/facilitators to partner notification may be different in the antenatal care context (as opposed to an STI clinic, for example) would be helpful – in the introduction, paragraph 4 the concern about the ‘health of the baby’ is mentioned; while this seems likely to be true, is there literature or formative work to suggest this will increase motivations for partner notification? Is there data to support the statement that more of these women will be in stable relationships? In some settings, pregnancy can be a higher risk time for intimate partner violence – is there any data on this from Zimbabwe? What resources will be available to women who report social harms from partner notification?
- Will study participants in the control arm be asked whether their partners were notified and sought treatment elsewhere? The authors mention that the free partner treatment at the study site(s) may motivate partners to present there for care, but if they have to pay for transport they may choose to go somewhere closer. It seems not asking about treatment sought elsewhere among the control partners could bias the results in favor of the intervention.
- Why was 28 days chosen as the window for partner treatment in the primary outcome?
- The secondary outcomes include uptake of PN slips by pregnant women – will this be compared between the arms? Earlier in the methods it states that participants will only be informed of the availability of an incentive once they test positive on an incentive day and have already informed the clinical team how many PN slips are required – will they be allowed to request more PN slips after learning of the intervention?
- In the discussion, the important issue of partner notification in the context of asymptomatic screening (in a setting where syndromic management is standard of care) is raised – this is an important issue and the discussion could be expanded and also supported with literature (see for example Hansman et al. Intl Jrnl STD AIDS 2021 https://pubmed.ncbi.nlm.nih.gov/34304619/)
- In the limitations, can you state what key differences between urban and rural settings in a country such as Zimbabwe would make the results less generalizable to rural settings? (eg, distance to travel to clinic; socioeconomic differences changing the value of the incentive, or others?)

**Minor comments: **

- Introduction, paragraph 3 mentions a “ *Systematic review of partner services in sub-Saharan Africa found…*” – this is missing a reference
- Introduction, paragraph 7 mentions a study in Malawi using a social contact recruitment program and $2 financial incentive – would be helpful to mention the outcome of this study.
- In the abstract and the study aim statement in the last paragraph of the introduction, the outcome is stated as “improving PN” or “improving uptake of PN” – however more concretely the goal is to assess the impact of the financial incentive on completion of partner treatment (as opposed to only notifying partners who do not then present for treatment) - recommend being more specific in these statements.
- Randomization section: consider using more scientific/neutral terminology in place of “gaming the system” which could be perceived as judgmental or dismissive of the potential complex motivations for an individual to do so.

Is the study design appropriate for the research question?

Yes

Is the rationale for, and objectives of, the study clearly described?

Yes

Are sufficient details of the methods provided to allow replication by others?

Yes

Are the datasets clearly presented in a useable and accessible format?

Not applicable

Reviewer Expertise:

Implementation science; epidemiology; sexually transmitted infections

I confirm that I have read this submission and believe that I have an appropriate level of expertise to confirm that it is of an acceptable scientific standard, however I have significant reservations, as outlined above.

## References

[1] : Experiences and preferences with sexually transmitted infection care and partner notification in Gaborone, Botswana. *Int J STD AIDS* .2021;32(13) : 10.1177/09564624211033231 1250-1256 10.1177/09564624211033231 34304619
